# Blood lead levels among children aged 6–30 months: An electronic health record–based study in Southern California, 2016–2025

**DOI:** 10.1016/j.envres.2026.125525

**Published:** 2026-08-20

**Authors:** Casey Morgan Luc, Gloria C. Chi, Rudy Patrick, Vicki Y. Chiu, Matt Feaster, Sara Y. Tartof

**Affiliations:** a Epidemic Intelligence Service, Division of Workforce Development, Centers for Disease Control and Prevention, Atlanta, GA, USA; b Department of Research & Evaluation, Kaiser Permanente Southern California, Pasadena, CA, USA; c Division of Epidemiology and Disease Control, Public Health Department, Pasadena, CA, USA

## Abstract

**Background:**

Lead exposure can cause long-term developmental deficits in children. We examined associations between sociodemographic characteristics and blood lead testing and levels in Southern California, where environmental lead hazards might exacerbate blood lead risk.

**Methods:**

We conducted a population-based analysis using repeated annual observations among Kaiser Permanente Southern California (KPSC) members aged 6–30 months from January 1, 2016–September 30, 2025 (with 2025 observations available through September only) to assess trends over time. Measured exposures included individual- and neighborhood-level sociodemographic characteristics. Outcomes included receipt of blood lead testing and blood lead levels (BLLs) ≥3.5 μg/dL. Modified Poisson regression with generalized estimating equations was used to estimate adjusted prevalence ratios (aPRs), accounting for age, sex, race and ethnicity, county, Medi-Cal status, neighborhood deprivation index (NDI), and year.

**Results:**

Among 923,144 KPSC annual records from 529,869 unique children, testing was relatively stable over time, with a modest ~1% annual decline (aPR, 0.99; 95% CI: 0.98–0.99). Testing was more common among Medi-Cal recipients and children residing in higher NDI quartiles. Among 324,291 total tests (277,361 unique children), 0.7% of tests had BLLs ≥3.5 μg/dL (n = 2,134), and prevalence declined over time (aPR, 0.91; 95% CI: 0.88–0.94). BLLs ≥3.5 μg/dL were lower among Hispanic children compared with non-Hispanic White children, with variation observed by Medi-Cal status and NDI.

**Conclusions:**

Blood lead testing rates changed little over time, whereas BLLs ≥3.5 μg/dL declined. However, differences in BLL ≥3.5 μg/dL persisted across sociodemographic groups, and a clinically important number of children continued to have elevated levels. Ongoing strategic prevention efforts remain important to reduce childhood lead exposure in Southern California.

## 1. Introduction

During 2011–2018, blood lead levels (BLLs) among children in the United States significantly declined, largely because of federal policies and coordinated public health interventions at both federal and state levels (Centers for Disease Control and Prevention). However, routine blood lead testing among children declined sharply across the country during the early stages of the COVID-19 pandemic. Although testing rates have rebounded, they have done so unevenly across regions (Kaufman et al., 2024). In California, for instance, childhood blood lead testing rates have improved postpandemic but remain approximately 20% below prepandemic levels (Centers for Disease Control and Prevention). Prior studies have shown that disruptions in preventive pediatric services, such as blood lead screening, disproportionately affected Medicaid-enrolled and other socioeconomically disadvantaged populations, suggesting a disproportionate recovery in testing rates across groups (Kaufman et al., 2024; Courtney et al., 2021; Batioja et al., 2023).

In California, blood lead testing is required at 12 and 24 months of age for children enrolled in publicly funded programs such as Medi-Cal, California's Medicaid program. Testing is also required for children who live in or frequently visit a home or building constructed before 1978, when lead-based paint was banned for residential use, that has peeling or chipped paint or has been recently remodeled (California Department of Public Health). Testing is recommended in certain other situations, including suspected lead exposure, at parental request, or for children who have recently immigrated from countries with high levels of environmental lead (California Department of Public Health). Nationwide, undertesting of children enrolled in Medicaid and other vulnerable children has been documented (Knighton et al., 2016; Department of Health and Human Services Office of Inspector General). Undertesting hinders the early identification of lead exposure sources, raising concern given the increased risk among children in California for BLLs ≥3.5 μg/dL, where environmental lead hazards, such as contaminated soil, are described as pervasive throughout the state (California Department of Public Health). While some other states implement universal screening with a goal to improve case detection by identifying asymptomatic children without known risk factors, it increases testing volume and resource use, whereas California's risk-based approach, supplemented by required screening for Medi-Cal-enrolled children, prioritizes higher-risk populations but may miss children with unrecognized exposures (Centers for Disease Control and Prevention; Council On Environmental H. Prevention of Childhood Lead Toxicity, 2016; McMenamin et al., 2018).

In October 2021, the Centers for Disease Control and Prevention (CDC) lowered its blood lead reference value from 5 μg/dL to 3.5 μg/dL, ensuring public health action to prevent long-term adverse health effects of lead poisoning in children earlier (Centers For Disease Control and Prevention). Importantly, even low levels of lead in the blood can produce irreversible health effects (Centers for Disease Control and Prevention). Lead exposure is particularly harmful in the early development of children, where it can cause developmental delays, cognitive impairments, and other lifelong challenges (Centers for Disease Control and Prevention). Recent 2022 state-level data show that elevated BLLs among children younger than 6 years vary across California, with generally lower rates observed in local health jurisdictions serving Southern California (California Department of Public Health). To the best of our knowledge, no studies have evaluated sociodemographic correlates to explain these differences in BLLs ≥3.5 μg/dL within these local health jurisdictions. Additionally, the January 2025 wildfires in Los Angeles County, among the most destructive in the state's history, raised concerns about the potential release and redistribution of environmental contaminants, including lead, further emphasizing the importance of evaluating sociodemographic and geographic correlates of vulnerability to exposure in this population. (County of Los Angeles; County of Los Angeles Public Health).

Because sociodemographic and neighborhood-level differences in childhood lead exposure are well documented (Hanna-Attisha et al., 2016; Hauptman et al., 2021; Jacobs and Brown, 2023), electronic health records (EHR) can provide valuable insights into the burden of lead exposure in specific communities. Prior studies using surveillance, administrative, or ecological data have often lacked individual-level clinical detail or relied on geographically aggregated measures, which may limit concurrent evaluation of temporal trends in testing patterns and BLLs, as well as sociodemographic correlates (Zartarian et al., 2024; Cantor et al., 2019). An EHR-based analysis offers a more granular view of BLLs at the community level and also includes demographic information that might not be captured in federal, state and local health department reports (Ghildayal et al., 2024; Parums, 2025; Weiskopf and Weng, 2013). This can help identify differences and guide more effective, prioritized lead control efforts at the local level. Therefore, we sought to describe trends and correlates of blood lead testing and levels among children receiving care in Southern California to aid primary prevention efforts.

## 2. Methods

This population-based analysis using repeated annual observations was conducted at Kaiser Permanente Southern California (KPSC), a large, integrated health care delivery system serving approximately 4.9 million members across the region, which includes Imperial, Kern, Los Angeles, Orange, Riverside, San Bernardino, San Diego, and Ventura Counties. Members are enrolled through commercial, federal, or state insurance plans. Members are sociodemographically diverse and generally representative of the underlying population in Southern California (Koebnick et al., 2012; Davis et al., 2023). The study population was restricted to children aged 6–30 months who were eligible if they had ≥6 months of continuous enrollment during a calendar year. Each calendar year meeting the enrollment criterion was included as a separate child-year observation. The testing analysis included all eligible child-year observations regardless of whether a blood lead test was performed, whereas blood lead outcome analyses were restricted to child-year observations with at least one blood lead test. We focused on children aged 6–30 months because of evidence of increased susceptibility to lead exposure from hand-to-mouth behavior and vulnerability to illness during early growth and development (Centers for Disease Control and Prevention; Mayans, 2019).

Blood lead concentrations were measured at College of American Pathologists (CAP)-accredited and Clinical Laboratory Improvement Amendments (CLIA)-certified KPSC regional laboratories using a NexION 2000 (PerkinElmer) inductively coupled plasma mass spectrometry (ICP-MS), an acceptable standard for accurate, confirmatory measurement of BLLs under CLIA regulations and CDC guidelines (Centers for Disease Control and Prevention). The same ICP-MS platform, standardized analytical procedures, and laboratory reporting conventions were used across KPSC regional laboratories throughout 2016–2025. During the study period, blood lead specimens were collected almost exclusively by venipuncture; capillary collection was not implemented until March 2025, and specimen source was not consistently documented thereafter. Consequently, reliable counts of capillary specimens were unavailable. Elevated results underwent repeat analysis of the original specimen for analytical verification. For elevated results from specimens submitted in micro-guard tubes, the laboratory report additionally recommended collection of a new venous specimen for confirmatory testing. The reference range was ≤3.4 μg/dL. The analytical sensitivity (limit of detection) and functional sensitivity of the assay were 0.105 μg/dL and 0.720 μg/dL, respectively, and the reportable range was 2.0–150.0 μg/dL. Results exceeding 70.0 μg/dL were designated as critical values. Quality assurance and quality control procedures were maintained throughout the study period. These included analysis of three levels of quality control material (BioRad Lyphochek Whole Blood Control Level 1 #561, Level 2 #562, and Level 3 #563) with each analytical batch, daily instrument calibration with a single element lead calibration standard (PerkinElmer N93000175), participation in external proficiency testing three times per year, routine instrument correlation studies for blood lead testing, and scheduled preventive maintenance performed according to manufacturer requirements.

An elevated BLL was defined as ≥3.5 μg/dL, consistent with the updated blood lead reference value recommendation for children by the California Department of Public Health (CDPH) and CDC(Centers For Disease Control and Prevention; California Department of Public Health). Aligned with CDC childhood blood lead surveillance methodology, (Centers for Disease Control and Prevention) children were counted once per calendar year based on their highest BLL. For children with multiple blood lead tests within a calendar year, the highest blood lead value was retained for analysis. Children could contribute one observation per calendar year and were included in multiple years if tested during more than one calendar year.

Blood lead testing results were obtained from EHR using Current Procedural Terminology code 83655. Date of birth, sex, race and ethnicity, county of residence, and Medi-Cal as insurance payer were obtained from KPSC membership administrative records. Race and ethnicity were categorized as follows: Hispanic, non-Hispanic (NH) Asian/Pacific Islander, NH Black, NH White, and other, multiple, unknown race or ethnicity. The neighborhood deprivation index (NDI), a composite measure based on results from the American Community Survey summarizing the economic disadvantage of individual census tracts (Messer et al., 2006), was also obtained. Assignment of NDI was based on a member's census tract of residence. Higher NDI values indicate greater neighborhood deprivation. Annual testing percentages were calculated using all eligible child-year observations as the denominator and child-year observations with a blood lead test as the numerator.

Descriptive statistics were calculated among children who completed ≥1 blood lead test. Annual observed percentages of testing and BLLs ≥3.5 μg/dL were calculated as crude proportions with 95% confidence intervals based on binomial distributions.

For adjusted analyses, we used modified Poisson regression models to estimate adjusted prevalence ratios (aPRs) and 95% confidence intervals (CIs) for factors associated with receipt of blood lead testing among all children aged 6–30 months. To estimate factors associated with BLL ≥3.5 μg/dL, we fit a separate modified Poisson regression model restricted to tested children.

All models included calendar year, age, sex, race and ethnicity, Medi-Cal status, NDI and county of residence as covariates. Calendar year was modeled as a continuous predictor after exploratory analyses found no evidence of meaningful nonlinearity. The unit of analysis was the child-year (eligible annual observation), with the child serving as the clustering unit for repeated observations across years. Generalized estimating equations with an exchangeable correlation structure were used to account for within-child correlation across repeated observations and assumed a common correlation among repeated measurements from the same child. Regression estimates represent population-averaged effects. Robust (sandwich) variance estimators were used to obtain valid standard errors (Zou, 2004). Modified Poisson regression modeling has been used previously to evaluate factors associated with elevated BLLs among children (Shakya and Bhatta, 2019). Missing data were minimal (<0.01%), and complete-case analyses were performed. Multicollinearity was assessed using pairwise correlations among predictors (|r| ≥ 0.70). No evidence of multicollinearity was observed. Adjusted temporal trends were derived from modified Poisson regression models with calendar year modeled as a continuous variable. Model-based predicted annual prevalence estimates were generated with covariates held constant at their reference categories and are presented alongside observed annual trends. All statistical analyses were conducted using SAS software, version 9.4 (SAS Institute Inc., Cary, NC).

This study was reviewed by CDC and was conducted consistent with applicable federal law and CDC policy — 45 C.F.R. part 46, 21 C.F.R. part 56; 42 U.S.C. Sect. 241(d); 5 U.S.C. Sect. 552a; 44 U.S.C. Sect. 3501 et seq. The KPSC Institutional Review Board reviewed and approved this study.

## 3. Results

During 2016–September 30, 2025, we identified a total of 923,144 KPSC records for 529,869 unique children aged 6–30 months. A total of 277,361 children completed ≥1 blood lead test with a total of 324,291 tests completed (Supplemental Fig. 1). Testing proportions remained relatively stable during the study period, except for declines during 2020 (28.7%) and 2025 (31.5%) (Supplemental Fig. 2). Testing was highest in Orange County (38.5%) and Los Angeles County (37.0%), and lowest in Ventura County (22.0%) (Supplemental Fig. 3). Median annual number of children tested was 33,496 children (range: 27,285–35,312 children). Most participants who received ≥1 blood test were aged 6–12 months, male, Hispanic and resided in Los Angeles (Table 1). Approximately a third (28.8%) received Medi-Cal. Among Medi-Cal enrollees, 43.7% received a lead screening test, higher than 32.3% of non-Medi-Cal members who received a lead screening test. Imperial County (56.9%, n =29) and Kern County (40.8%, n =2,821) had the highest proportions of children in the most vulnerable NDI quartile; whereas, Ventura County (8.0%, n = 361) and Orange County (10.7%, n = 3,687) had the lowest.

In adjusted analyses to determine factors associated with testing among all 923,144 KPSC records, blood lead testing prevalence was 31% higher among those who received Medi-Cal, compared with those who did not (aPR, 1.31; 95% CI: 1.30–1.32) (Table 2). Compared with NH White children, testing prevalence was 31% higher among NH Asian/Pacific Islander children (aPR, 1.31; 95% CI: 1.29–1.32) and 19% higher among Hispanic children (aPR, 1.19; 95% CI: 1.18–1.20). Compared with the lowest NDI quartile (least deprived), blood lead testing prevalence was higher in each successively higher quartile, with the highest testing prevalence in the highest NDI quartile (most deprived) (aPR, 1.19; 95% CI: 1.19–1.21). Compared with Los Angeles County, testing prevalence was 32% lower in Ventura County (aPR; 0.68; 95% CI: 0.66–0.70). The prevalence of testing in the overall population decreased by approximately 1% per year over the study period (aPR, 0.99; 95% CI: 0.98–0.99), consistent with the largely stable trend observed in Supplemental Fig. 2.

Among the 324,291 blood lead tests, 316,682 (97.7%) were reported at the lower reporting limit of 2.0 μg/dL, while 7,609 (2.4%) had values > 2.0 μg/dL. Observed values ranged from the lower reporting limit of 2.0 to 62.2 μg/dL. Overall, 2,134 (0.7%) resulted in BLLs ≥3.5 μg/dL (Table 3). These results occurred among 1,856 unique children, of whom 1,597 had one BLL ≥3.5 μg/dL test, 240 had two, and 19 had three. Among the 259 children with two or more BLLs ≥3.5 μg/dL, the largest proportion (n = 101; 39.0%) resided in the highest NDI quartile (most deprived). Overall, proportion of BLLs ≥3.5 μg/dL declined over time in our sample, from 0.8% in 2016 to 0.5% in 2025 (Fig. 1). The highest proportion of BLLs ≥3.5 μg/dL were reported in Ventura County (0.84% [42 total cases]) and in Los Angeles County (0.83% [1,208 total cases]), with 1 BLL ≥3.5 μg/dL case in Imperial County accounting for 1.79% of the 56 tests completed (Fig. 2). Despite the similar BLL ≥3.5 μg/dL proportion, there were more cases overall in Los Angeles County (n = 1,208), compared with Ventura County (n = 42).

Table 4 summarizes results from the adjusted modified Poisson regression model estimating aPRs for BLLs ≥3.5 μg/dL. The prevalence of BLL ≥3.5 μg/dL decreased over time, with each additional calendar year associated with a 9% lower prevalence of BLL ≥3.5 μg/dL (aPR, 0.91; 95% CI: 0.88–0.94), consistent with the observed declining trend in Fig. 1. Compared with the unadjusted model, adjustment for demographic and clinical characteristics attenuated several year-specific associations, with prevalence ratios moving closer to the null for some years; however, lower prevalence of BLL ≥3.5 μg/dL remained in later years. In the adjusted model, the prevalence ratio for BLL ≥3.5 μg/dL comparing the highest versus lowest NDI quartile was 1.47 (95% CI: 0.96–2.25) (Table 4). Among children aged 6–30 months, each additional 6 months of age was associated with an 18% lower prevalence of BLL ≥3.5 μg/dL (aPR, 0.82; 95% CI: 0.76–0.89). Compared with NH White children, prevalence of BLL ≥3.5 μg/dL among Hispanic children was 38% lower (aPR, 0.62; 95% CI: 0.48–0.80). Children with Medi-Cal had a higher prevalence of BLL ≥3.5 μg/dL compared with those without Medi-Cal (aPR, 1.27; 95% CI: 0.98–1.66). Compared with Los Angeles County residents, Riverside County residents had a 53% lower prevalence of BLL ≥3.5 μg/dL (aPR, 0.47; 95% CI: 0.31–0.71).

## 4. Discussion

Among a large, demographically and geographically diverse sample of children aged 6–30 months in Southern California, the proportion receiving blood lead testing remained relatively stable during the study period (2016–2025). Despite a slight reduction in testing rates during 2020 and 2025, trends in BLLs ≥3.5 μg/dL remained generally consistent over the study period. During 2016–2025, BLLs ≥3.5 μg/dL declined continuously by year among tested children, with a 0.5% prevalence of BLLs ≥3.5 μg/dL in 2025.

The observed decline in BLLs ≥3.5 μg/dL may reflect multiple, non-mutually exclusive factors. These include potential reductions in childhood lead exposure associated with ongoing lead hazard control and prevention efforts (Dignam et al., 2019), changes in the demographic composition of the population over time, (United States Census Bureau) or changes in testing patterns that influence which children undergo blood lead screening (Hauptman et al., 2021). The 2021 CDC blood lead reference value update may have increased detection of elevated BLLs after 2021, suggesting that the observed decline is unlikely to be attributable to this change. Although testing rates remained relatively stable overall, blood lead testing was not performed uniformly across the population and varied by socioeconomic, clinical, and healthcare utilization factors. Consequently, temporal trends may reflect changes in the underlying population being tested in addition to changes in lead exposure.

Efforts by local health departments, including Los Angeles County Department of Public Health and the City of Pasadena Public Health Department to offer prevention guidance and community-based blood lead testing may have supported ongoing lead awareness and screening activities; however, the present study was not designed to evaluate the effectiveness of these programs or their contribution to observed trends (City of Pasadena, 2025). Nonetheless, childhood lead exposure remains an important public health concern. More than 1800 tested children in the study population had BLLs ≥3.5 μg/dL during the study period, indicating a clinically important burden of exposure. Given evidence that even low-level lead exposure can adversely affect neurodevelopment and that no safe BLL has been identified, (Centers for Disease Control and Prevention) continued surveillance, screening, and primary prevention efforts remain warranted.

Although declines in childhood BLLs have been previously reported (Egan et al., 2021), notable differences in testing rates and BLLs were observed. Testing was more frequent among those in the most deprived NDI quartiles and children enrolled in Medi-Cal, the state's Medicaid program that provides health care for low-income residents. Blood lead testing is mandated for Medi-Cal beneficiaries, and the higher testing rates observed among children in the most deprived NDI quartiles likely reflect greater engagement in screening among populations with higher social and healthcare-related risk factors for lead exposure. However, despite higher testing rates, the prevalence of BLL ≥3.5 μg/dL remained slightly higher among children in the most deprived NDI quartiles, although the adjusted CI included the null. Changes in BLLs are likely a result of source elimination through remediating lead hazards in homes or education to avoid lead-contaminated products, rather than being attributable to expanded testing itself. Screening alone does not reduce BLLs but may facilitate identification of children who require follow-up and exposure mitigation. Nevertheless, the higher testing rates observed in these communities suggest that screening efforts are reaching children in populations with greater potential risk for lead exposure, although available data do not include key environmental risk factors needed to determine whether screening is reaching children with the greatest biological exposure risk. Furthermore, efforts are underway at CDPH to expand blood lead prevention efforts through partnerships with state and local organizations. This includes participation in community events, resulting in 3543 community outreach activities completed during 2021–2022 (California Department of Public Health). Continued investment in initiatives that extend beyond testing may continue to reduce lead exposure and promote early identification of BLLs ≥3.5 μg/dL in the most at-risk children.

Findings from the adjusted prevalence ratio analyses demonstrated pronounced differences across groups: prevalence of BLL ≥3.5 μg/dL was lowest among Hispanic and NH Asian/Pacific Islander children compared to NH White children. Secondly, rates of testing and BLLs were similar between NH Black and NH White children. Lead screening programs among NH Black and NH White children who are Medi-Cal eligible, when coupled with lead hazard remediation and educational outreach, may support earlier identification of children with elevated BLLs and facilitate exposure reduction efforts.

Additionally, differences were observed in testing rates and BLLs across counties. Overall, Kern, Ventura, and Los Angeles Counties had the highest prevalence of BLLs ≥3.5 μg/dL. However, testing rates were lowest in Kern and Ventura Counties despite their relatively high prevalence of BLLs ≥3.5 μg/dL.

The essential role of EHR data, especially within integrated prepaid healthcare delivery systems (e.g., KPSC) is also evident because these data allow for the monitoring of blood lead trends while adjusting for demographic and clinical characteristics. By leveraging EHR data, public health officials and health organizations may gain insights into BLL ≥3.5 μg/dL trends and help guide lead control activities more effectively.

This analysis has certain limitations. First, there is potential for missing data on lead testing conducted outside the KPSC system. However, requiring at least 6 months of continuous KPSC membership likely reduced the extent of missing data, as continuously enrolled children are more likely to receive routine clinical care and have laboratory testing captured within the system. Secondly, the sample used for the BLL ≥3.5 μg/dL modeling may differ from the blood lead testing sample, as testing prevalence was higher among children enrolled in Medi-Cal. However, the larger proportion of Medi-Cal enrolled children in the BLL ≥3.5 μg/dL modeling likely reflects a population at increased risk for BLLs ≥3.5 μg/dL. Third, blood lead testing may not be performed uniformly across the KPSC population and may vary by clinical and socioeconomic factors. Measurement variability between capillary and venous samples may have introduced some misclassification of BLLs, although nearly all samples were venous. Completion of recommended venous confirmation for elevated capillary results could not be consistently verified in the available EHR data. In addition, because testing is not random and is influenced by socioeconomic and clinical factors such as Medi-Cal status and NDI, the analytic sample may not be fully representative of all children in the population. Changes over time in healthcare utilization, membership composition, insurance coverage, care-seeking behaviors, or the capture of laboratory results within the EHR may also have influenced observed temporal trends. Therefore, declines in BLLs ≥3.5 μg/dL among tested children should not be interpreted solely as evidence of declining exposure in the underlying population. Additionally, because data for 2025 were available only through September 30, estimates for 2025 represent a partial-year observation and may not be directly comparable with complete calendar years. However, sensitivity analyses excluding 2025 yielded similar overall temporal patterns for both testing and BLL outcomes, suggesting that the observed trends were not driven solely by the inclusion of partial-year 2025 data (Supplemental Table 1). Fourth, although individual-level socioeconomic data were not available, the use of county of residence, Medi-Cal status membership, and NDI as proxies for socioeconomic status likely provided a reasonable approximation of these factors. However, several important determinants of childhood lead exposure were absent from these analyses, including housing age and condition, immigration or refugee status, parental occupation, industrial proximity, water source, and nutritional or iron deficiency status. Finally, the geographic analyses are descriptive and reflect the proportion of tested children with BLLs ≥3.5 μg/dL rather than population prevalence. The analyses do not incorporate formal spatial modeling, uncertainty estimates, or population-standardized comparisons, and estimates for counties with small numbers of tested children should be interpreted cautiously, which limits the ability to draw definitive conclusions about spatial patterns of BLLs. Despite these limitations, the study benefits from the large, diverse KPSC cohort, which allows for reliable estimations of blood lead testing rates and BLLs in the last decade.

In summary, the declining rates of children with BLLs ≥3.5 μg/dL observed in this analysis are promising. However, prevalence of BLLs ≥3.5 μg/dL among certain populations and the overall persistence of BLLs ≥3.5 μg/dL, even at low levels, must continue to be monitored, because no level of lead exposure is considered safe (Centers for Disease Control and Prevention). The use of EHRs within managed care organizations may be useful for tracking trends, examining associations with sociodemographic factors, and informing public health responses to reduce lead exposure risks.

## Supplementary Material

Supplemental Figure 1

Supplemental Figure 2

Supplemental Table 1

Supplemental Figure 3

## Figures and Tables

**Fig. 1.**
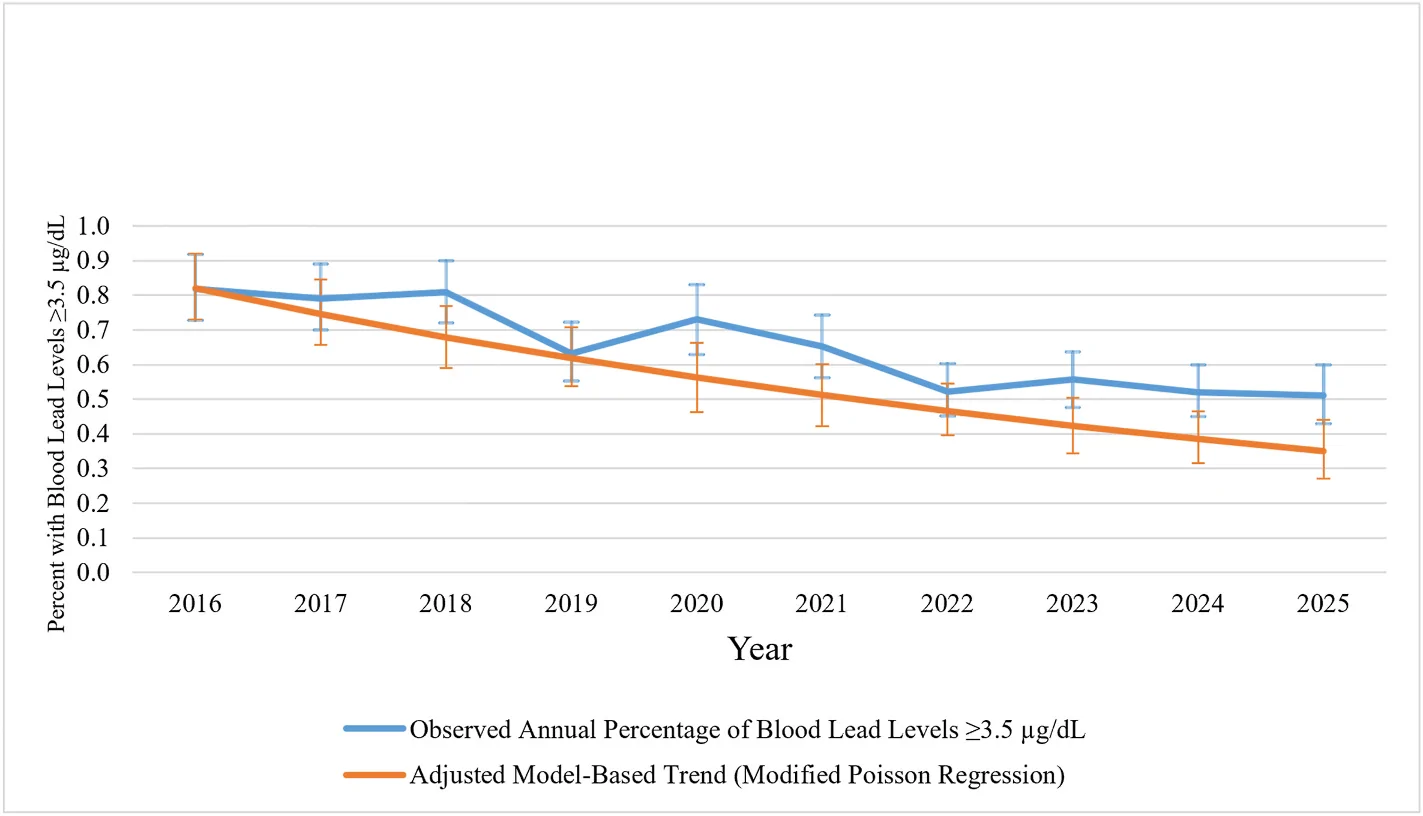
Annual Prevalence of Blood Lead Tests with Blood Lead Levels ≥3.5 μg/dL Among Children Aged 6–30 Months, Kaiser Permanente Southern California, 2016–2025 (n = 2,134 Blood Lead Levels ≥3.5 μg/dL; N = 324,291 Tests).

**Fig. 2.**
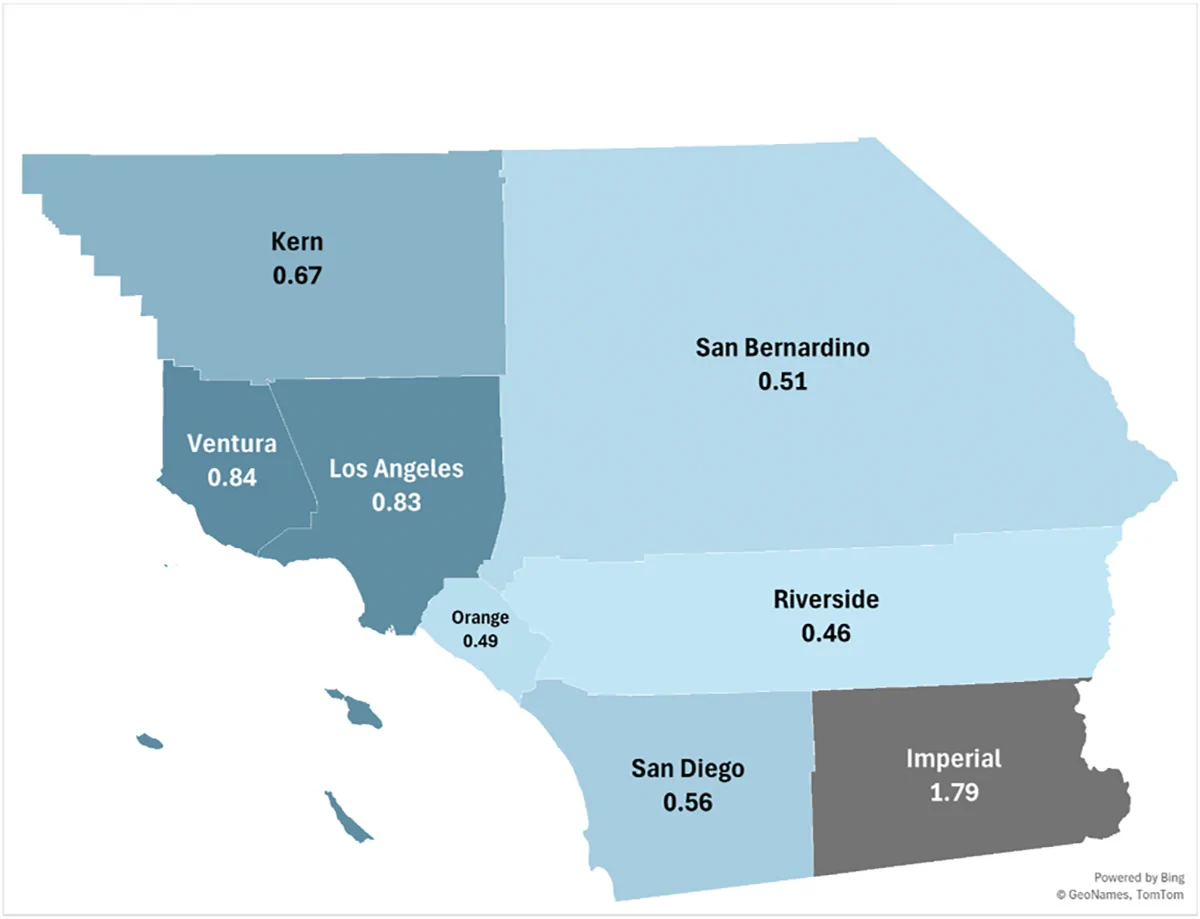
Test Positivity Percentage of Blood Lead Levels ≥3.5 μg/dL Among Children Aged 6–30 Months, Kaiser Permanente Southern California, 2016–2025 (n = 2,134 Blood Lead Levels ≥3.5 μg/dL; N = 324,291 Tests) Percentages represent the proportion of tested child-year observations with BLL ≥3.5 μg/dL and should not be interpreted as population prevalence. Estimates for counties with small denominators, including Imperial County (n = 56 tests), should be interpreted cautiously.

**Table 1:** Demographic characteristics of tested children aged 6–30 months, Kaiser Permanente Southern California, 2016–2025.

Characteristic^a^	≥1 Blood Lead Test(s), N (%), N = 277,361

**Age**	
6–12 Mo	112,097 (40.4)
>12–18 Mo	100,680 (36.3)
>18–24 Mo	42,246 (15.2)
>24–30 Mo	22,338 (8.1)
**Sex**	
Female	135,063 (48.7)
Male	142,298 (51.3)
**Race/Ethnicity**	
Hispanic	147,776 (53.3)
Non-Hispanic (NH) Asian/Pacific Islander	35,505 (12.8)
NH Black	17,525 (6.3)
NH White	61,058 (22.0)
Other/Multiple Race/Ethnicity	15,497 (5.6)
**County**	
Imperial	51 (0.02)
Kern	6,917 (2.5)
Los Angeles	125,256 (45.2)
Orange	36,638 (13.2)
Riverside	37,480 (13.5)
San Bernardino	34,466 (12.4)
San Diego	32,054 (11.6)
Ventura	4,499 (1.6)
**Medi-Cal Status** ^b^	
Yes	79,818 (28.8)
No	197,543 (71.2)
**Neighborhood Deprivation Index**	
Lowest 25%, Least Deprived	72,044 (26.0)
>25%–50%	71,713 (25.9)
>50%–<75%	69,694 (25.1)
Highest 25%, Most Deprived	63,903 (23.0)
Missing	7 (<0.01)

a Characteristics from earliest blood lead test between 2016 and 2025. Values, such as county residence or Medi-Cal status, might be different in subsequent tests among those with >1 blood lead test in the follow-up period.

b Medi-Cal is the name of California's Medicaid program.

**Table 2:** Adjusted Associations Between Demographic Characteristics and Blood Lead Testing Among Children Aged 6–30 Months, Kaiser Permanente Southern California, 2016–2025, with Complete Data (N = 923,110 Records; ^a^ n = 529,869 Children).

Characteristic	Adjusted Prevalence Ratio (95% CI)

**Age (per 6 months)**	0.81 (0.81–0.82)
**Female (ref = Male)**	1.00 (0.99–1.00)
**Race/Ethnicity**	
Hispanic	1.19 (1.18–1.20)
Non-Hispanic (NH) Asian/Pacific Islander	1.31 (1.29–1.32)
NH Black	1.02 (1.01–1.03)
NH White	1.00
Other/Multiple Race/Ethnicity	0.95 (0.94–0.97)
**Medi-Cal Status (ref = No)**	1.31 (1.30–1.32)
**County**	
Imperial	0.99 (0.82–1.20)
Kern	0.77 (0.76–0.79)
Los Angeles	1.00
Orange	1.11 (1.10–1.11)
Riverside	1.04 (1.03–1.05)
San Bernardino	0.86 (0.85–0.87)
San Diego	0.94 (0.93–0.95)
Ventura	0.68 (0.66–0.70)
**Neighborhood Deprivation Index**	
Lowest 25%, Least Deprived	1.00
>25%–50%	1.07 (1.06–1.08)
>50%–<75%	1.12 (1.11–1.13)
Highest 25%, Most Deprived	1.19 (1.19–1.21)
**Calendar Year**	0.99 (0.98–0.99)

a 34 observations did not have complete data for adjusted analysis and were removed.

**Table 3:** Proportion of blood lead level tests exceeding Selected thresholds among children aged 6–30 months, Kaiser Permanente Southern California, 2016–2025, N = 324,291 ^a^.

Blood Lead Level (BLL) Threshold (μg/dL)	Tests ≥ Threshold, N (%)

> 2.0 (Lower Reporting Limit)	7,609 (2.4)
≥ 3.0	3,063 (0.9)
≥3.5 (Elevated BLL)	2,134 (0.7)
≥ 5.0	952 (0.3)
≥ 10.0	228 (0.1)

a Children with repeated blood lead testing contributed multiple observations to the descriptive distributions.

**Table 4:** Adjusted Associations Between Demographic Characteristics and Blood Lead Levels Among Children Aged 6–30 Months, Kaiser Permanente Southern California, 2016–2025, with Complete Data (N = 324,283 Tests; ^a^ n = 277,361 Children).

Characteristic	Elevated Blood Lead Level (≥3.5 μg/dL)	
	Adjusted Prevalence Ratio (95% CI)	*P*

**Age (per 6 months)**	0.82 (0.76–0.89)	<0.001
**Female (ref = Male)**	1.08 (0.91–1.29)	0.394
**Race/Ethnicity**		
Hispanic	0.62 (0.48–0.80)	<0.001
Non-Hispanic (NH) Asian/Pacific Islander	0.78 (0.58–1.03)	0.081
NH Black	0.95 (0.67–1.36)	0.787
NH White	1.00	-
Other/Multiple Race/Ethnicity	0.86 (0.58–1.28)	0.472
**Medi-Cal Status (ref = No)**	1.27 (0.98–1.66)	0.066
**County**		
Imperial	2.91 (0.42–20.27)	0.282
Kern	0.97 (0.55–1.70)	0.913
Los Angeles	1.00	-
Orange	0.76 (0.46–1.07)	0.114
Riverside	0.47 (0.31–0.71)	<0.001
San Bernardino	0.71 (0.48–1.04)	0.080
San Diego	0.84 (0.64–1.11)	0.227
Ventura	1.36 (0.86–2.16)	0.190
**Neighborhood Deprivation Index**		
Lowest 25%, Least Deprived	1.00	-
>25%–50%	1.28 (0.87–1.89)	0.203
>50%–<75%	1.21 (0.80–1.84)	0.358
Highest 25%, Most Deprived	1.47 (0.96–2.25)	0.075
**Calendar Year**	0.91 (0.88–0.94)	<0.001

a 8 observations did not have complete data for adjusted analysis and were removed.

## Data Availability

The data that has been used is confidential.

## Appendix A. Supplementary data

Supplementary data to this article can be found online at https://doi.org/10.1016/j.envres.2026.125525.
