# Supplementary material for: Blood lead levels among children aged 6–30 months: An electronic health record–based study in Southern California, 2016–2025
Source: Environ Res. Author manuscript; Available in PMC 2026 Oct 14. (PMC13667763; doi:10.1016/j.envres.2026.125525)

Supplemental Figure 3. Percentage of Completed Blood Lead Tests Among Children Aged 6–30 Months, Kaiser Permanente Southern California, 2016–2025 (n = 324,291 Tests; N = 923,144 Records)

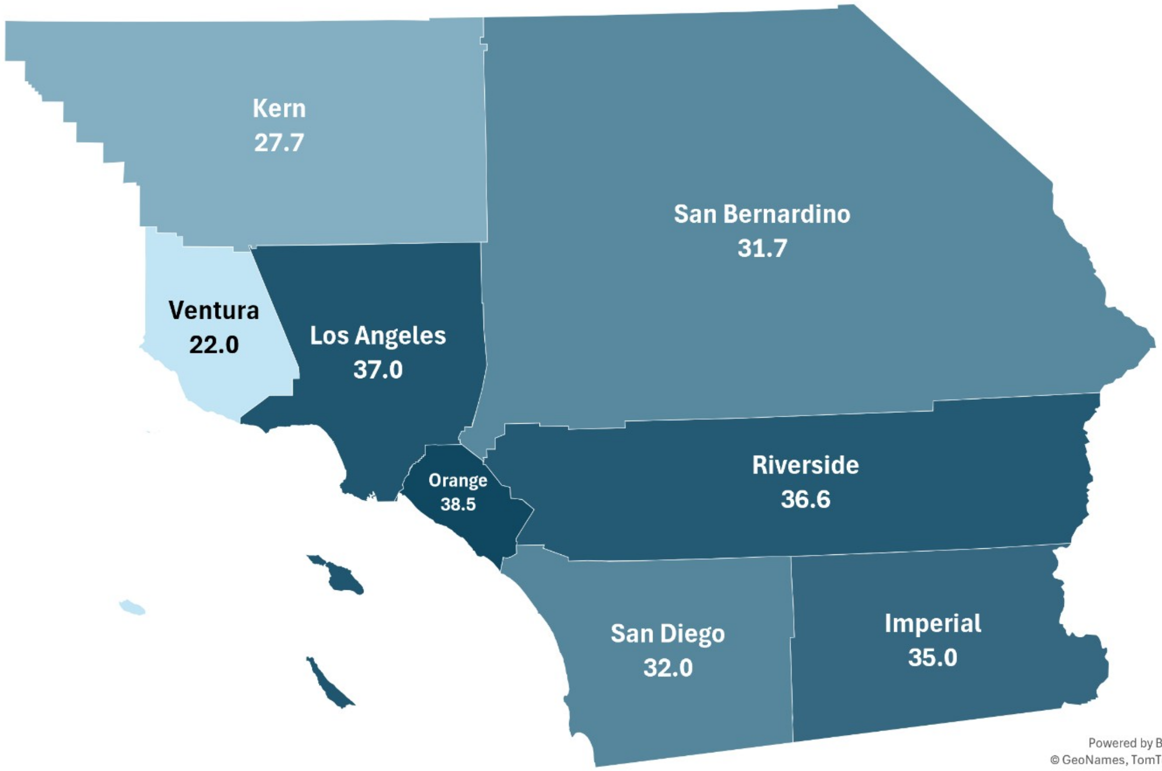

Supplement: Supplementary file 1 [file NIHMS2211020-supplement-Supplemental_Figure_3.docx]
