# Supplementary material for: Blood lead levels among children aged 6–30 months: An electronic health record–based study in Southern California, 2016–2025
Source: Environ Res. Author manuscript; Available in PMC 2026 Oct 14. (PMC13667763; doi:10.1016/j.envres.2026.125525)
Supplement: Supplementary file 2 [file NIHMS2211020-supplement-Supplemental_Figure_2.docx]

Supplemental Figure 2. Annual Prevalence of Completed Blood Lead Tests Among Children Aged 6–30 Months, Kaiser Permanente Southern California, 2016–2025 (n = 324,291 Tests; N = 923,144 Records)

Percent of Records with Completed Blood Lead Test

| Year | Observed Annual Percentage of Completed Blood Lead Tests | Adjusted Model-Based Trend (Modified Poisson Regression) |
| --- | --- | --- |
| 2016 | 38.4 | 38.35 |
| 2017 | 36.5 | 37.9 |
| 2018 | 37.3 | 37.46 |
| 2019 | 37.1 | 37.02 |
| 2020 | 28.7 | 36.59 |
| 2021 | 34.1 | 36.17 |
| 2022 | 34.6 | 35.75 |
| 2023 | 36.7 | 35.34 |
| 2024 | 36.4 | 34.93 |
| 2025 | 31.5 | 34.53 |
