# Supplementary material for: Blood lead levels among children aged 6–30 months: An electronic health record–based study in Southern California, 2016–2025
Source: Environ Res. Author manuscript; Available in PMC 2026 Oct 14. (PMC13667763; doi:10.1016/j.envres.2026.125525)
Supplement: Supplementary file 4 [file NIHMS2211020-supplement-Supplemental_Table_1.docx]

**Supplemental Table 1. Sensitivity Analysis of Adjusted Associations Between Demographic Characteristics and Blood Lead Testing and Elevated Blood Lead Levels Among Children Aged 6–30 Months, Kaiser Permanente Southern California, 2016–2024 (Excluding 2025)**

| **Characteristic** | **Blood Lead Testing,**  **N = 836,473 Records**  **n = 485,383 Children** | **Elevated Blood Lead Level (≥3.5 µg/dL),**  **N = 296,998 Tests**  **n = 255,827 Children** |
| --- | --- | --- |
|  | **Adjusted Prevalence Ratio (95% CI)** | **Adjusted Prevalence Ratio (95% CI)** |
| **Age (per 6 months)** | 0.80 (0.80–0.81) | 0.85 (0.80–0.92) |
| **Female (ref = Male)** | 1.00 (0.99–1.00) | 1.05 (0.93–1.17) |
| **Race/Ethnicity** |  |  |
| Hispanic | 1.19 (1.18–1.20) | 0.72 (0.60–0.86) |
| Non-Hispanic (NH) Asian/Pacific Islander | 1.31 (1.29–1.32) | 0.81 (0.65–0.99) |
| NH Black | 1.02 (1.01–1.04) | 0.93 (0.72–1.20) |
| NH White | 1.00 | 1.00 |
| Other/Multiple Race/Ethnicity | 0.96 (0.95–0.97) | 0.97 (0.74–1.27) |
| **Medi-Cal Status (ref = No)** | 1.29 (1.28–1.30) | 1.29 (1.10–1.53) |
| **County** |  |  |
| Imperial | 0.91 (0.73–1.12) | 3.20 (0.47–21.99) |
| Kern | 0.76 (0.75–0.78) | 0.82 (0.55–1.21) |
| Los Angeles | 1.00 | 1.00 |
| Orange | 1.12 (1.11–1.13) | 0.71 (0.56–0.89) |
| Riverside | 1.04 (1.04–1.05) | 0.55 (0.43–0.70) |
| San Bernardino | 0.87 (0.86–0.87) | 0.67 (0.52–0.85) |
| San Diego | 0.93 (0.92–0.94) | 0.80 (0.65–0.97) |
| Ventura | 0.68 (0.66–0.69) | 1.17 (0.79–1.71) |
| **Neighborhood Deprivation Index** |  |  |
| Lowest 25%, Least Deprived | 1.00 | 1.00 |
| >25%–50% | 1.07 (1.06–1.08) | 1.22 (0.90–1.64) |
| >50%–<75% | 1.13 (1.12–1.13) | 1.30 (0.96–1.75) |
| Highest 25%, Most Deprived | 1.20 (1.19–1.21) | 1.66 (1.24–2.22) |
| **Calendar Year** | 0.99 (0.99–1.00) | 0.91 (0.89–0.93) |
